# Supplementary material for: Network-based restoration strategies maximize ecosystem recovery
Source: Commun Biol. 2023 Dec 12;6:1256. doi: 10.1038/s42003-023-05622-3 (PMC10716433; doi:10.1038/s42003-023-05622-3)
Supplement: Supplementary file 2 — Supplementary Information [file 42003_2023_5622_MOESM2_ESM.pdf]

Supplementary information

Network-based restoration strategies maximize ecosystem recovery

Udit Bhatia[*1,2], *Sarth Dubey*[3], *Tarik C. Gouhier*[4], *Auroop R. Ganguly*[2]

[1]Discipline of Civil Engineering, Indian Institute of Technology, Gandhinagar, Gujarat, India-382355

[2]Sustainability and Data Sciences Lab, Department of Civil & Environmental Engineering, Northeastern University, Boston, MA 02115, USA

[3]Discipline of Computer Science & Engineering, Indian Institute of Technology, Gandhinagar, Gujarat, India-382355

[4]Department of Marine and Environmental Sciences, Marine Science Center, Northeastern University, Nahant, MA 01908, USA

[*]Correspondence to: bhatia.u@iitgn.ac.in

This file includes:

Tables S1 to S4

Figures S1 to S23

Tables

Sr. No.	Name	latitude	longitude	S	A	C	N
1	M_PL_003	-33.223	-70.267	61	0.694	0.090	0.192
2	M_PL_022	-34.167	-69.700	66	2.143	0.088	0.180
3	M_PL_060_04	-20.730	57.730	67	2.191	0.139	0.265
4	M_PL_060_07	-20.730	57.730	68	1.345	0.096	0.234
5	M_PL_060_06	-20.730	57.730	71	1.731	0.082	0.146
6	M_PL_040	18.354	-77.645	72	1.483	0.091	0.199
7	M_PL_041	15.519	-61.467	74	1.387	0.109	0.274
8	M_PL_030	8.933	-67.417	81	1.893	0.074	0.112
9	M_PL_012	28.128	-17.249	84	1.897	0.091	0.304
10	M_PL_060_05	-20.730	57.730	87	1.636	0.081	0.173
11	M_PL_072_04	-37.842	-58.359	94	2.760	0.111	0.311
12	M_PL_023	-33.000	-69.283	95	3.130	0.076	0.229
13	M_PL_072_02	-37.842	-58.359	96	1.909	0.082	0.226
14	M_PL_031	5.583	-61.717	97	1.021	0.066	0.123
15	M_PL_035	17.917	-76.192	97	0.590	0.081	0.257
16	M_PL_017	51.575	-2.590	104	3.160	0.151	0.428
17	M_PL_072_01	-37.842	-58.359	106	1.718	0.072	0.202
18	M_PL_002	-33.223	-70.227	107	1.488	0.071	0.154
19	M_PL_010	74.500	-20.500	107	2.452	0.194	0.352
20	M_PL_014	81.817	-71.300	110	2.793	0.076	0.257
21	M_PL_043	56.239	9.954	110	2.929	0.109	0.222
22	M_PL_058	42.297	3.235	113	2.531	0.123	0.280
23	M_PL_072_03	-37.842	-58.359	117	2.079	0.080	0.236
24	M_PL_019	-36.450	148.267	125	2.125	0.078	0.193
25	M_PL_009	68.350	18.500	142	4.917	0.086	0.154
26	M_PL_018	56.239	9.974	144	2.692	0.094	0.197
27	M_PL_034	-42.000	-73.583	154	4.923	0.094	0.250
28	M_PL_026	-0.500	-90.500	159	0.514	0.036	0.251
29	M_PL_029	-43.100	171.720	167	2.408	0.060	0.158
30	M_PL_028	-43.028	171.785	180	3.390	0.066	0.164

Table S1 Characteristics of the 30 real-world networks analyzed in this study are arranged in ascending order of network size. The names and geographical location of the networks refer to their IDs, and the latitude-longitude coordinates from https://www.web-of-life.es. The network attributes listed here include network size(S), asymmetry(A), connectance(C) and nestedness(N).

Sr. No.	S	A	C	N
1	100	0.75	0.15	0.1304
2	100	0.75	0.15	0.1494
3	100	0.75	0.15	0.1680
4	100	0.75	0.20	0.1750
5	100	0.75	0.20	0.1934
6	100	0.75	0.20	0.2186
7	100	0.25	0.15	0.1395
8	100	0.25	0.15	0.1532
9	100	0.25	0.15	0.1720
10	100	0.25	0.20	0.1806
11	100	0.25	0.20	0.2011
12	100	0.25	0.20	0.2231
13	100	0.50	0.10	0.0859
14	100	0.50	0.10	0.1014
15	100	0.50	0.10	0.1171
16	100	0.50	0.15	0.1296
17	100	0.50	0.15	0.1517
18	100	0.50	0.15	0.1718
19	100	0.50	0.20	0.1749
20	100	0.50	0.20	0.1966
21	100	0.50	0.20	0.2200
22	100	0.75	0.05	0.0354
23	100	0.75	0.05	0.0424
24	100	0.75	0.05	0.0519
25	100	0.75	0.10	0.0859
26	100	0.75	0.10	0.1001
27	100	0.75	0.10	0.1223

Table S2 Characteristics of the 27 synthetic mutualistic systems generated are listed here for the network attributes: network size(S), asymmetry(A), connectance(C) and nestedness(N).

		Degree - Random			Degree - Betweenness			Degree - Closeness		
		R	G	S	R	G	S	R	G	S
1-D	X	1.35E-34	1.53E-31	3.88E-32	0.074	0.024	0.196	0.844	0.682	0.844
	ST	8.56E-30	5.96E-22	1.03E-14	0.984	0.984	0.157	0.299	0.196	0.001
	P	1.0	1.0	0.956	1.0	1.0	1.0	0.9995	1.0	0.956
2-D	X	5.58E-42	4.15E-28	1.53E-31	0.682	0.433	0.596	0.157	0.956	0.433
	ST	0.157	0.299	0.0001	0.043	0.299	0.024	0.013	0.043	0.024
	P	0.196	0.009	0.196	0.512	0.433	0.433	0.909	1.0	0.767

Table S3 p-values for 2-sample Kolmogorov-Smirnov Test for the restoration strategies using normalized distributions of the three key criteria: abundance (X), persistence (P) and settling time (ST); simulated under 1-D and 2-D models, and subjected to random (R), generalist-preferred (G) and specialist-preferred (S) perturbations.

Sr. No.	Symbol	Description
1	P_i	Population density or abundance of plant species i
2	A_i	Population density or abundance of pollinator species i
3	$\partial P_i/\partial t$	rate of change of abundance of plant species i
4	$\partial A_i/\partial t$	rate of change of abundance of pollinator species i
5	$\alpha_i(=0.3)$	intrinsic growth rate of species i
6	$\beta_{ij};\ i \neq j(=0.0)$	interspecific competition between species i and j
7	$\beta_{ij};\ i = j(=1)$	intraspecific competition within species i
8	$h(=0.7)$	half saturation constant
9	γ_{ik}	strength of the mutualistic interaction for species i with its mutualistic partners k
10	ϵ_{ij}	equal to 1 if interaction exists between species i and j, otherwise 0
11	$\gamma_0(=1)$	Mutualistic interaction strength constant
12	D_i	Number of mutualistic partners of species i
13	$p(=0.5)$	Trade-off factor between interaction strength and number of interactions
14	$\mu(=10^{-4})$	Net incoming migration
15	P_{eff}	Single effective plant population that approximates the species-to-species variations
16	A_{eff}	Single effective pollinator population that approximates the species-to-species variations
17	$<\gamma_P>$	Unweighted plant average mutualistic interacting strength
18	$<\gamma_A>$	Unweighted plant average mutualistic interacting strength
19	x_i	Population density or abundance of projected species i (projected network may be plants or pollinators)
20	$B_i(=0.1)$	Net incoming migration of projected species i
21	$K_i(=5)$	Carrying capacity for logistic growth
22	$C_i(=1)$	Abundance threshold for negative growth when abundance is low (Allee effect)
23	A_{ij}	Weighted projection adjacency matrix (projected network may be plants or pollinators)
24	$D_i(=5), E_i(=0.9), H_j(=0.1)$	Coefficients for saturation of mutualistic interaction benefits
25	β_{eff}	Nearest-neighbor weighted degree

Table S4 Appendix of symbols and its description. Entries 1-18 and 19-25 belong to 2-D and 1-D Models respectively.

19 # Figures

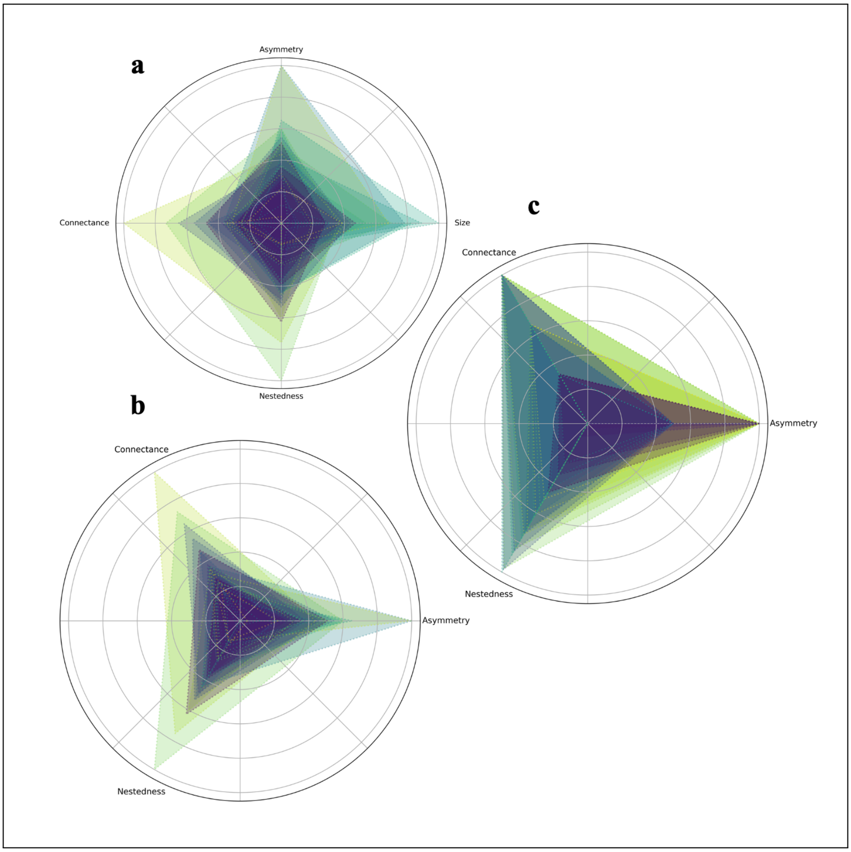

Fig. S1 Comparative Analysis of Network Attributes in Real-World and Synthetic Plant-Pollinator Networks (a) Radar plot showing the normalized network attributes (Network Size, Asymmetry, Connectance and Nestedness) for the 30 real-world plant-pollinator networks. The length of a spoke is proportional to the attribute's magnitude relative to its maximum magnitude. **(b)** Distribution of network attributes, excluding network size, of real-world networks and **(c)** Radar plot showing the network attributes of 27 (100 nodes/species) synthetically generated networks to study the spectrum of network structural attributes not captured in the 30 real-world networks.

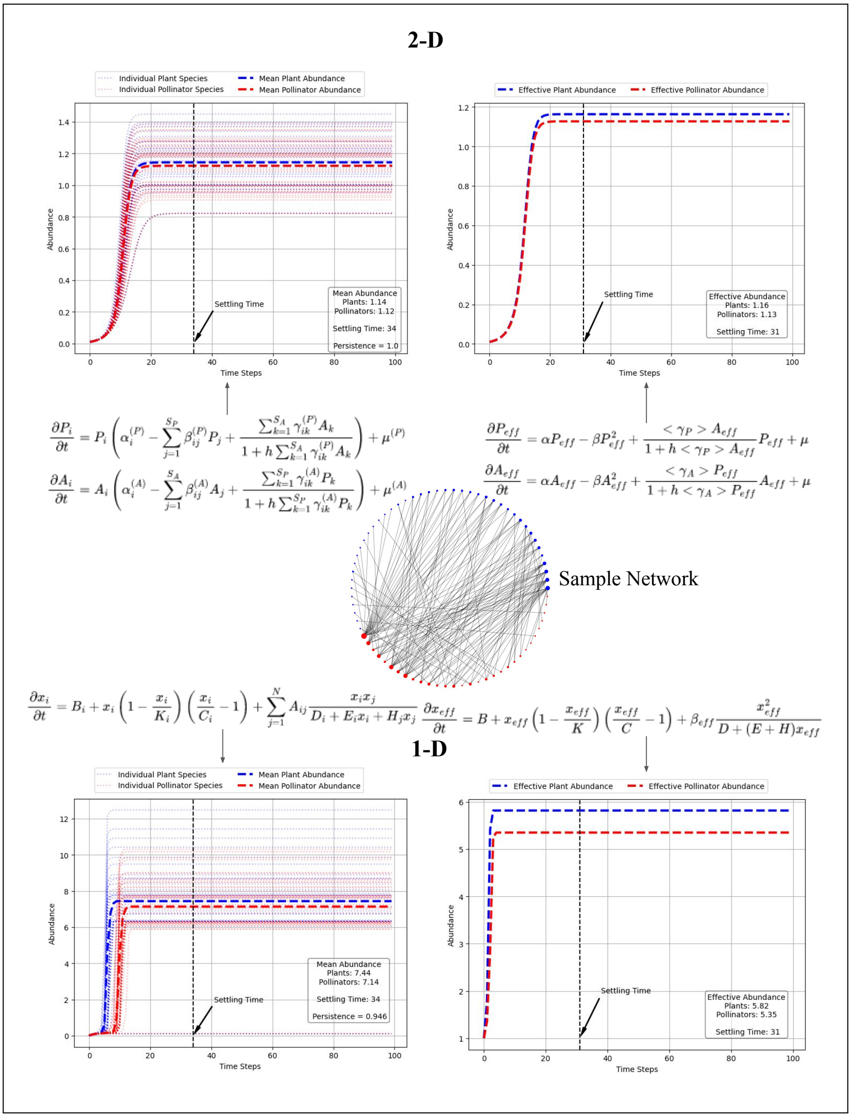

Fig. S2 The 1-D, 2-D and N-dimensional model response of the sample ecosystem (see 'M_PL_041' in Table S1) for the three key criteria.

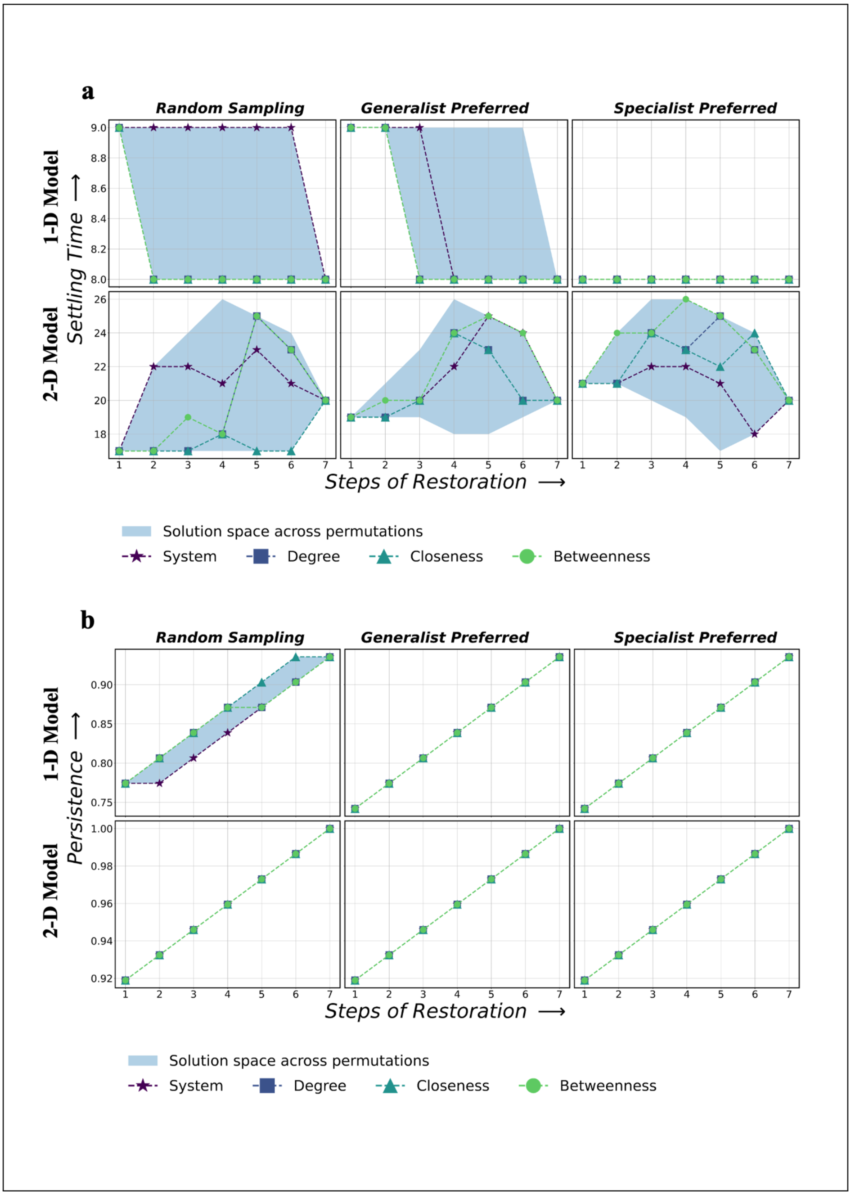

Fig. S3 An example ecosystem (see 'M_PL_041' in Table S1) is perturbed by removing 20% of the plant species (6 species). Outcomes of the two criteria - **(a)** settling time and **(b)** persistence for restoration strategies based on each topological property are plotted over the solution space generated by all possible reintroduction sequences.

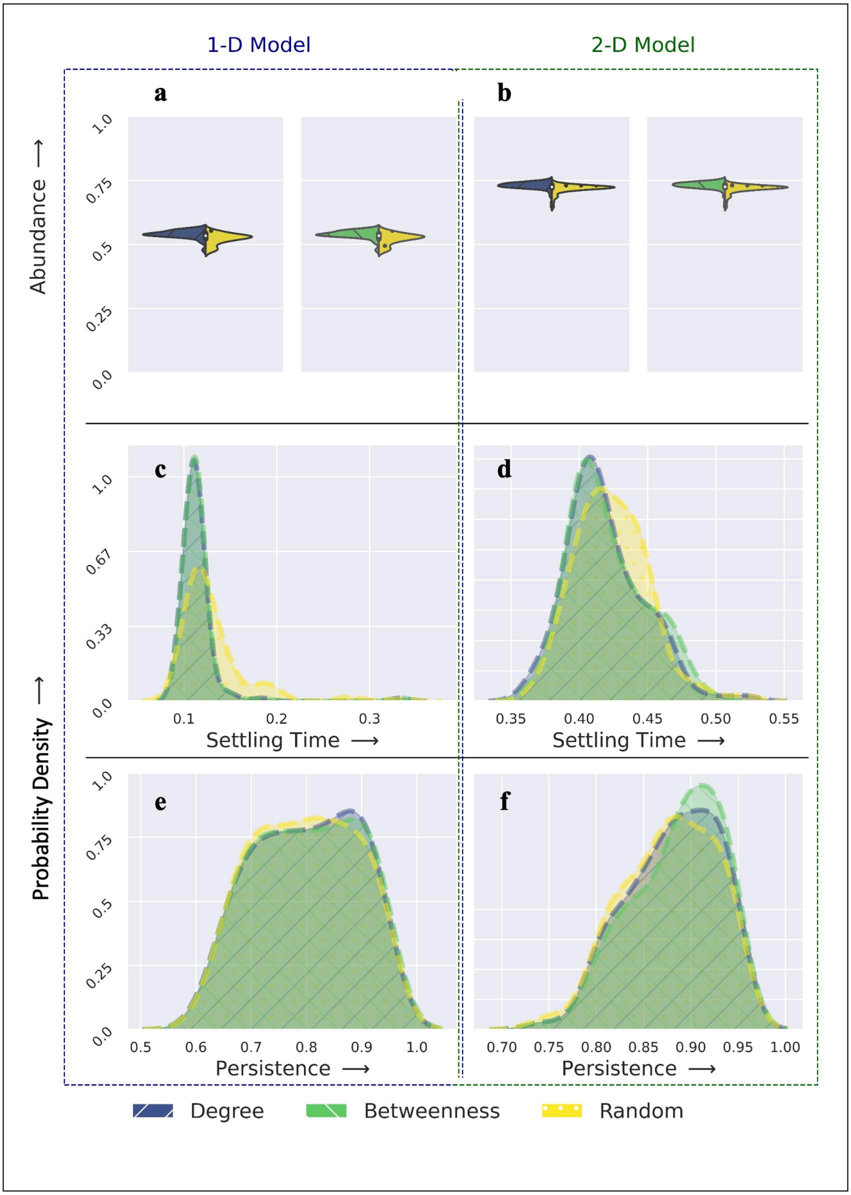

Fig. S4 Distributions of mean abundance X (**a**, **b**), settling time ST (**c**, **d**), and persistence P (**e**, **f**) for each restoration strategy under random perturbation scenarios where 30% of species were removed.

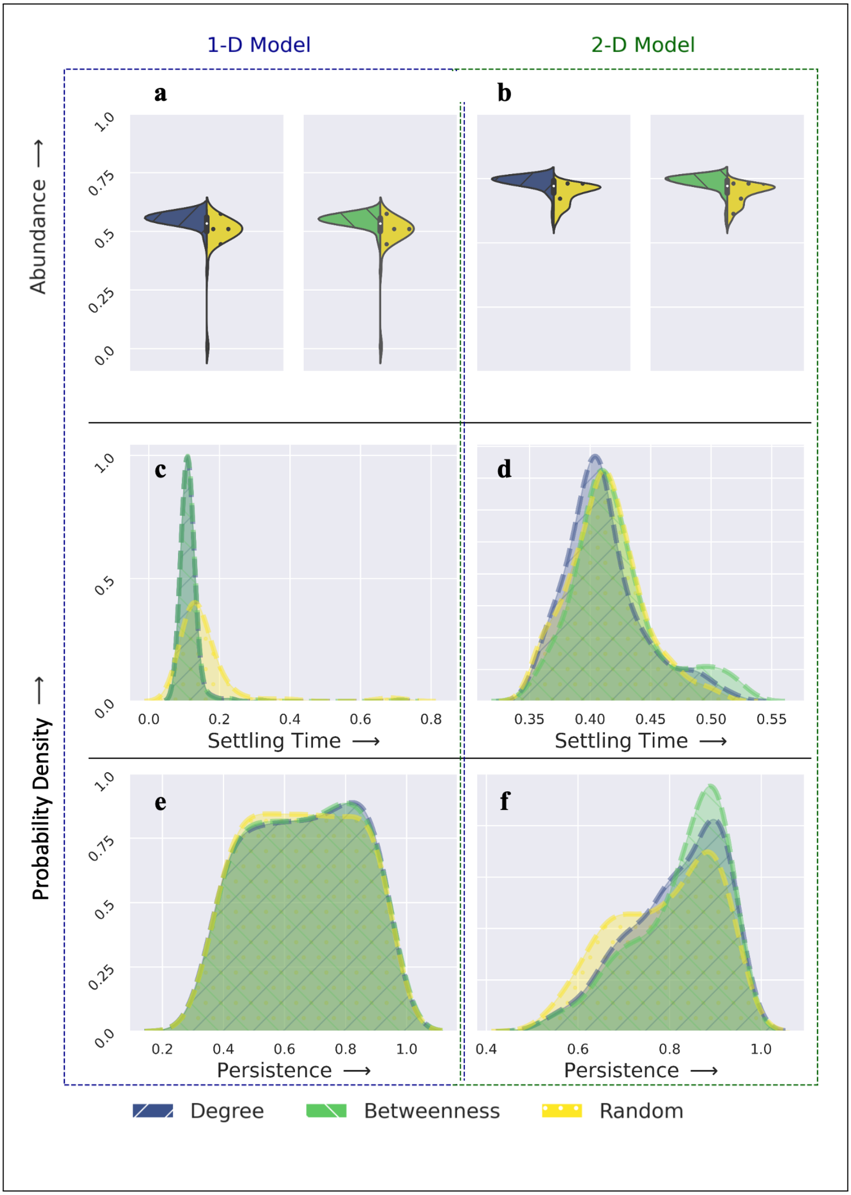

Fig. S5 Distributions of mean abundance X (**a**, **b**), settling time ST (**c**, **d**), and persistence P (**e**, **f**) for each restoration strategy under random perturbation scenarios where 60% of species were removed.

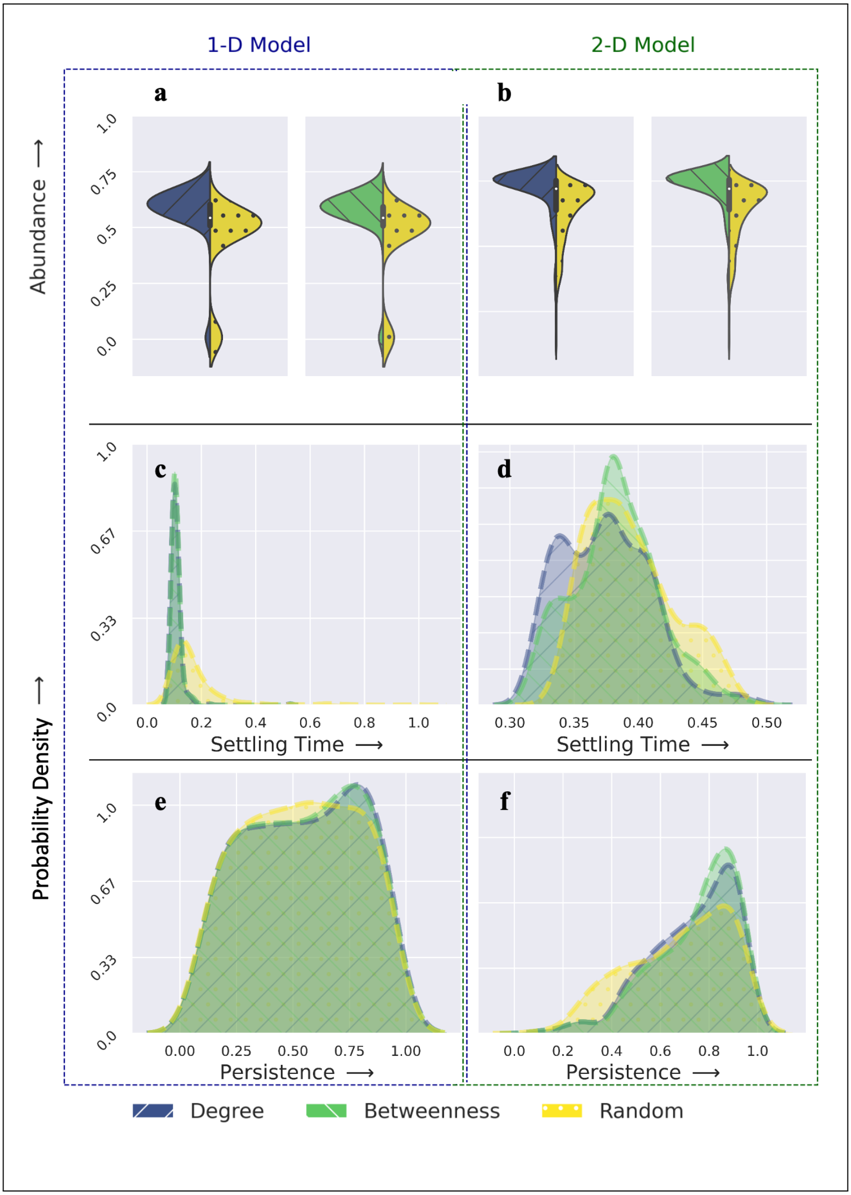

Fig. S6 Distributions of mean abundance X (**a**, **b**), settling time ST (**c**, **d**), and persistence P (**e**, **f**) for each restoration strategy under random perturbation scenarios where 90% of species were removed.

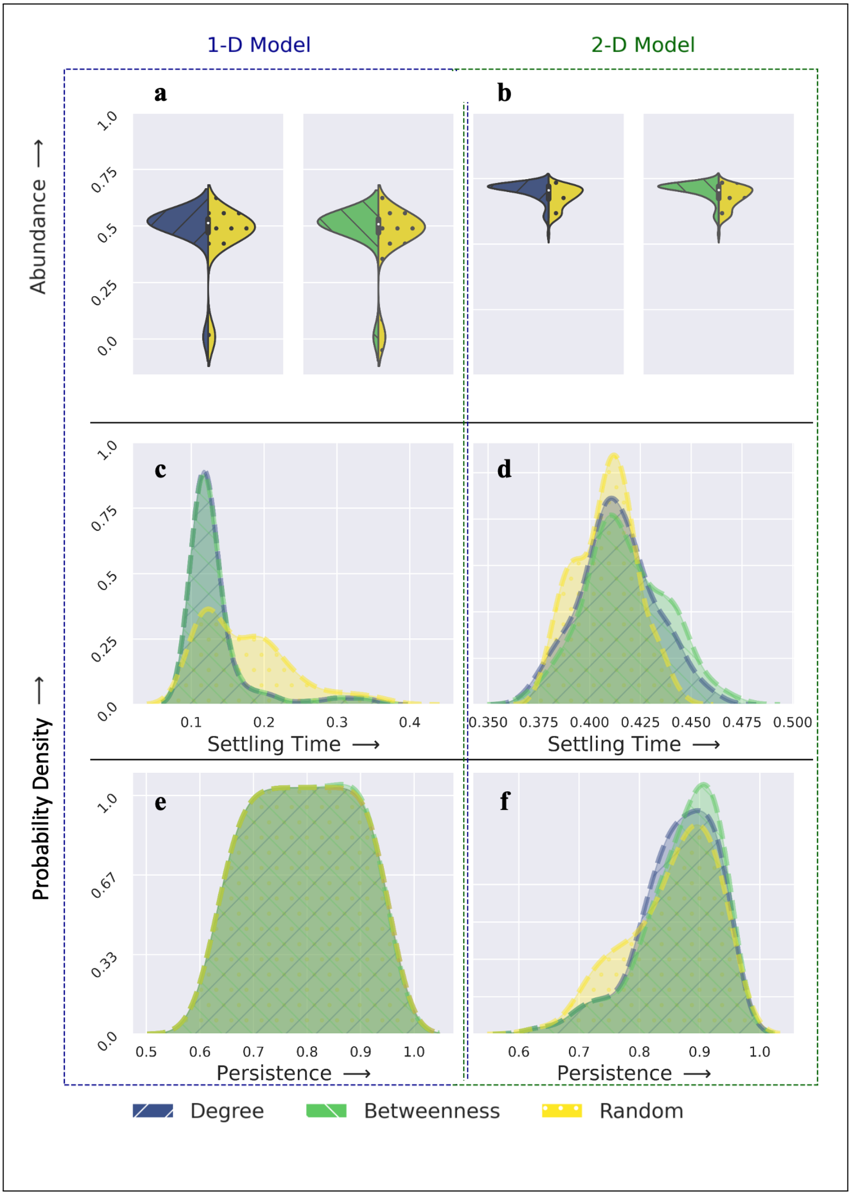

Fig. S7 Distributions of mean abundance X (**a**, **b**), settling time ST (**c**, **d**), and persistence P (**e**, **f**) for each restoration strategy under generalist-preferred perturbation scenarios where 30% of species were removed.

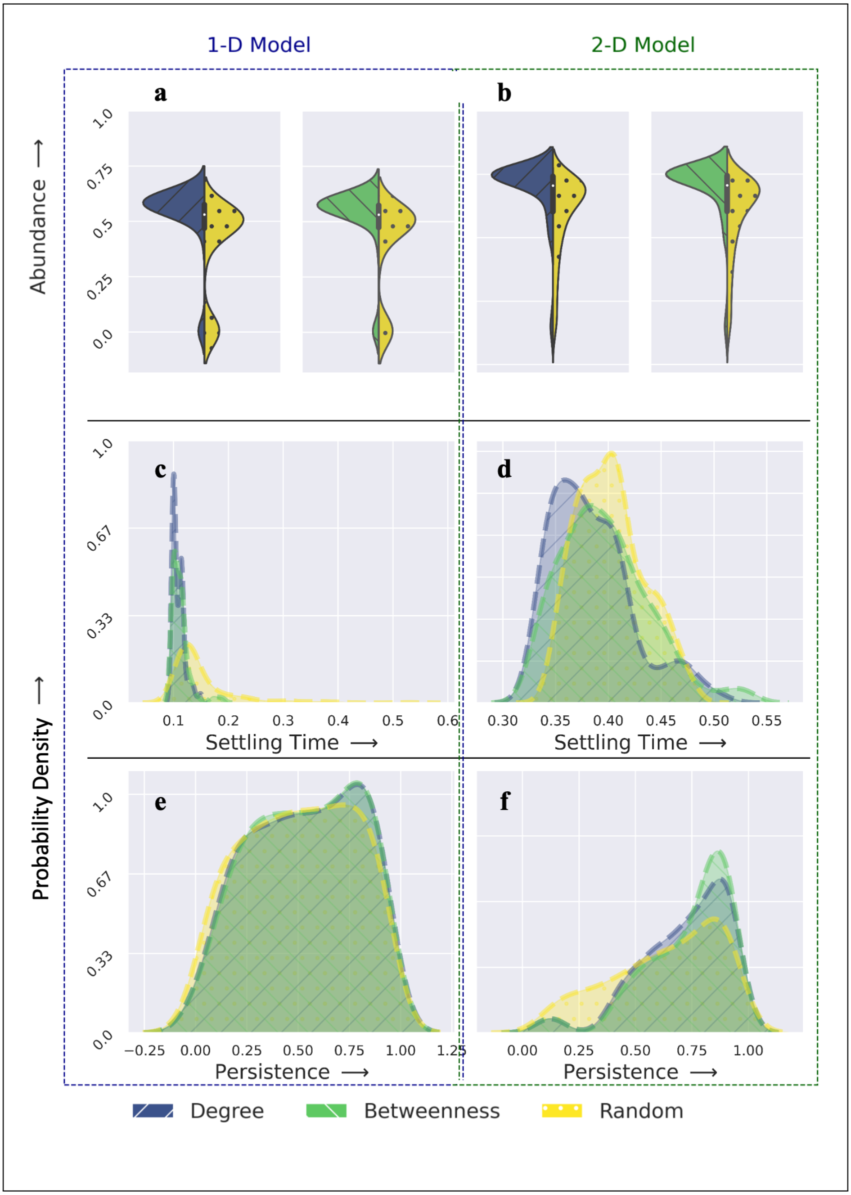

Fig. S8 Distributions of mean abundance X (**a**, **b**), settling time ST (**c**, **d**), and persistence P (**e**, **f**) for each restoration strategy under generalist-preferred perturbation scenarios where 90% of species were removed.

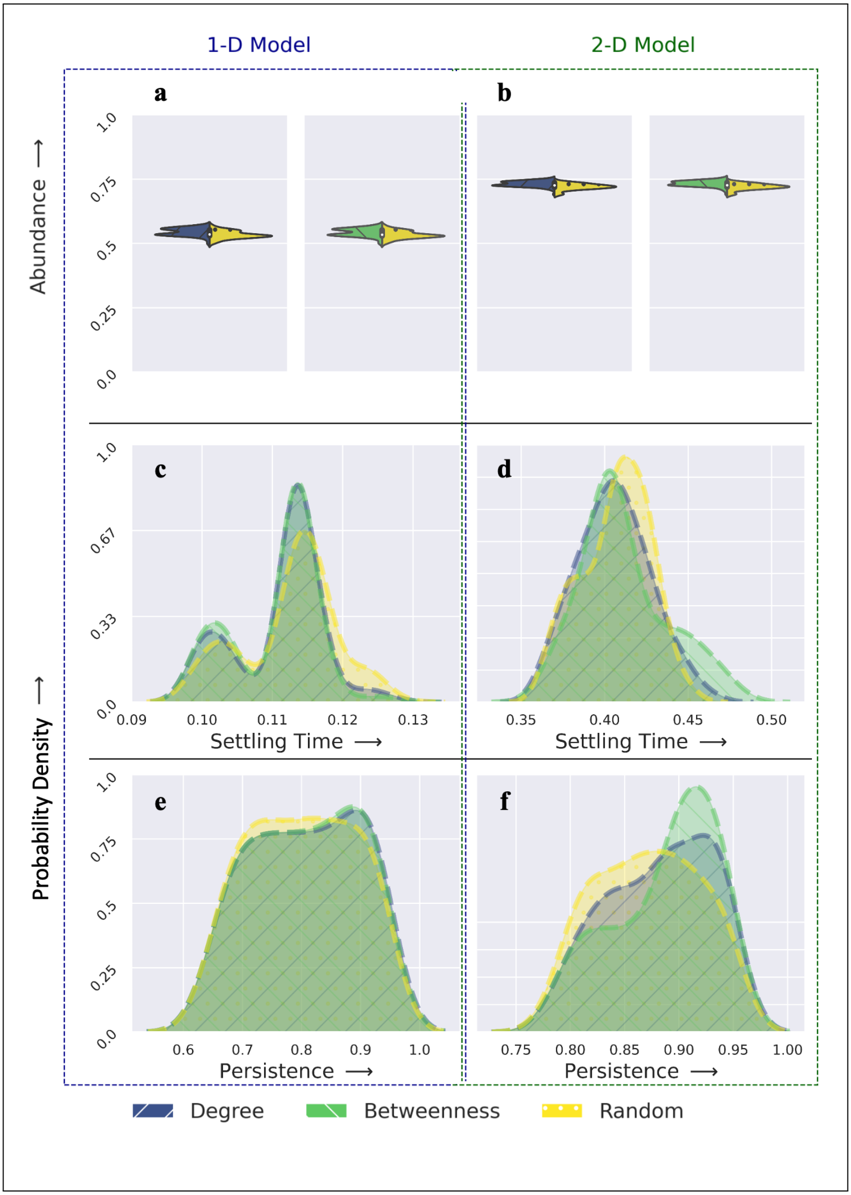

Fig. S9 Distributions of mean abundance X (**a**, **b**), settling time ST (**c**, **d**), and persistence P (**e**, **f**) for each restoration strategy under specialist-preferred perturbation scenarios where 30% of species were removed.

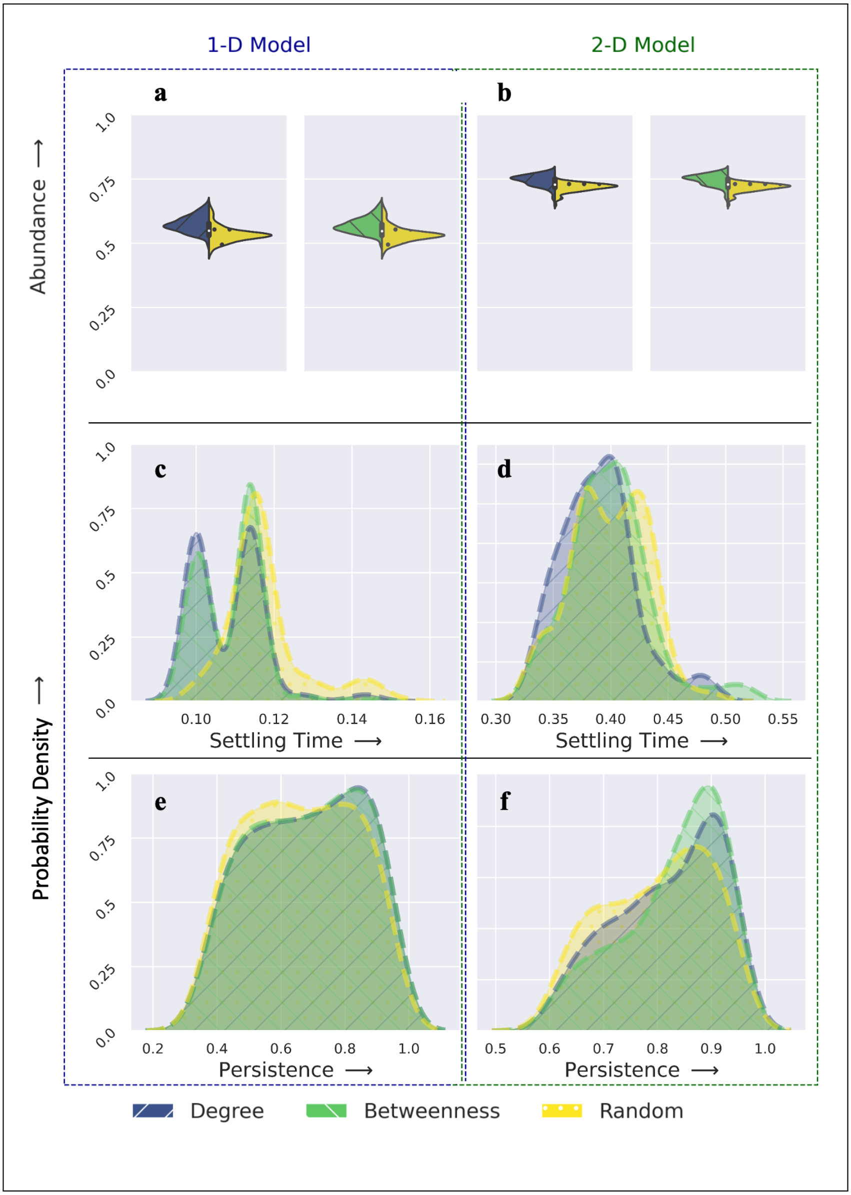

Fig. S10 Distributions of mean abundance X (**a**, **b**), settling time ST (**c**, **d**), and persistence P (**e**, **f**) for each restoration strategy under specialist-preferred perturbation scenarios where 60% of species were removed.

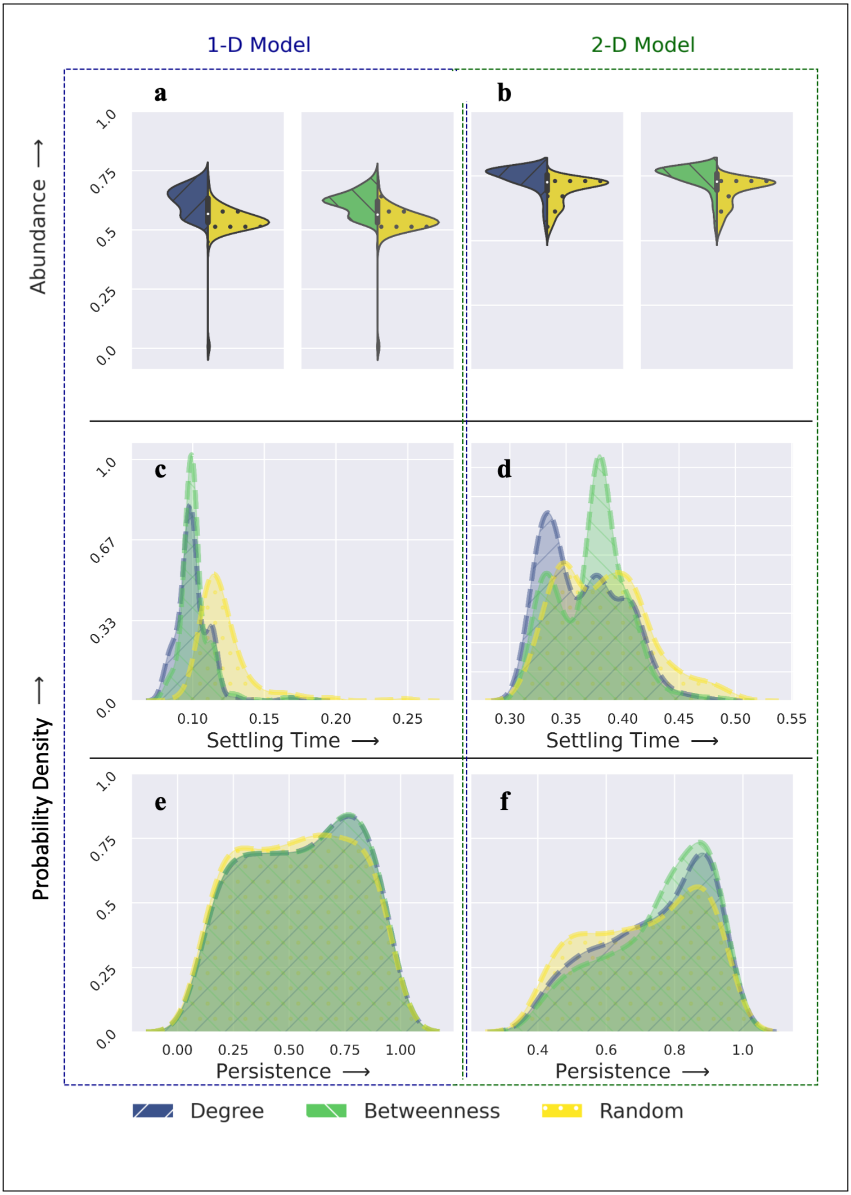

Fig. S11 Distributions of mean abundance X (**a**, **b**), settling time ST (**c**, **d**), and persistence P (**e**, **f**) for each restoration strategy under specialist-preferred perturbation scenarios where 90% of species were removed.

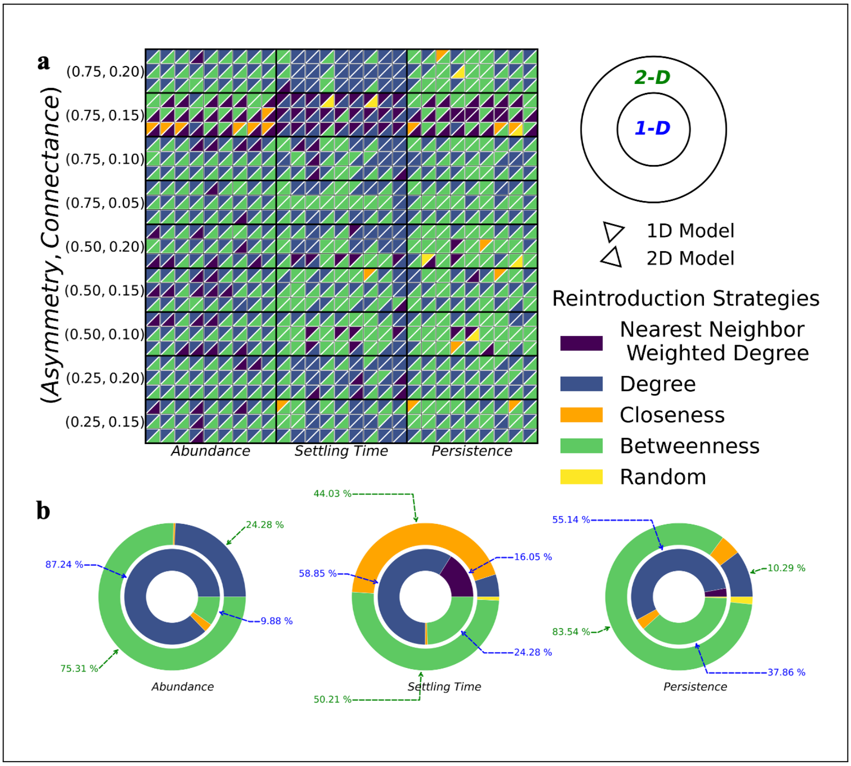

Fig. S12 Assessment of Restoration Strategies in Synthetic Plant-Pollinator Networks: (a) For the 27 synthetic networks studied with the three criteria (blocks with 9 columns each) and all nine perturbation scenarios: random selection of species (columns 1-3), generalist-preferred (columns 4-6) or specialist-preferred (columns 7-9), with perturbation corresponding to the removal of 30%, 60%, and 90% species (ordered left to right for each perturbation scenario). Restoration strategies are ranked based on a 'best-vs-rest' policy for 1-D and 2-D models (upper and lower triangle respectively). **(b)** The distribution of outperforming restoration strategies is drawn in a nested pie chart (1-D and 2-D models on inner and outer regions respectively) for each criterion.

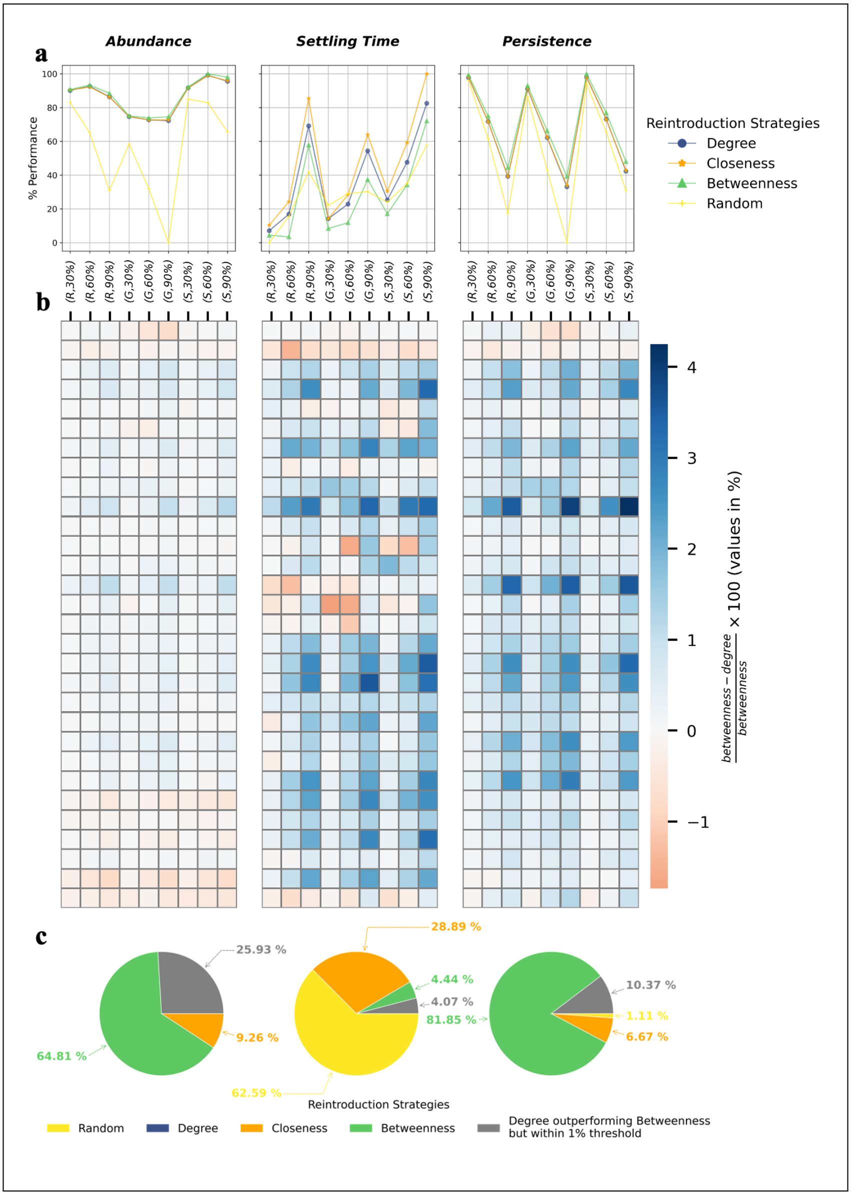

Fig. S13 Comparative Evaluation of Restoration Strategies in real-world ecosystems with respect to betweenness: (a) The performance of the restoration strategies measured for the three key criteria and all nine perturbation scenarios in a sample ecosystem (see 'M_PL_041' in Table S1). **(b)** This analysis is extended for the 30 real-world networks to show the relative performance of degree-based interventions compared to betweenness-based interventions. **(c)** The distribution of outperforming strategies when the improvement by degree-based interventions is within 1% of betweenness-based interventions.

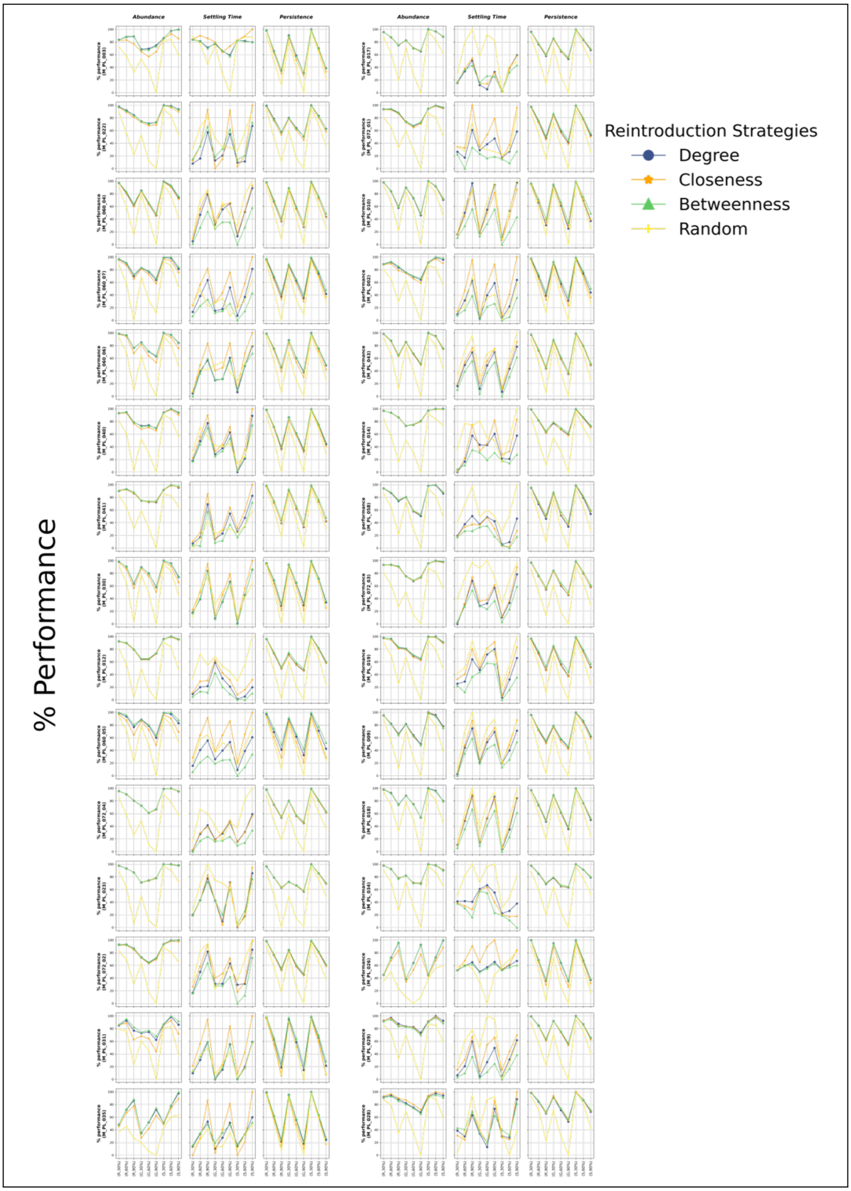

Fig. S14 The performance of the restoration strategies measured for the three key criteria and all nine perturbation scenarios is shown for all 30 real-world networks. For each of the three criteria, the restoration gains can be represented as % ranging from the lowest to highest performance across the nine perturbation scenarios.

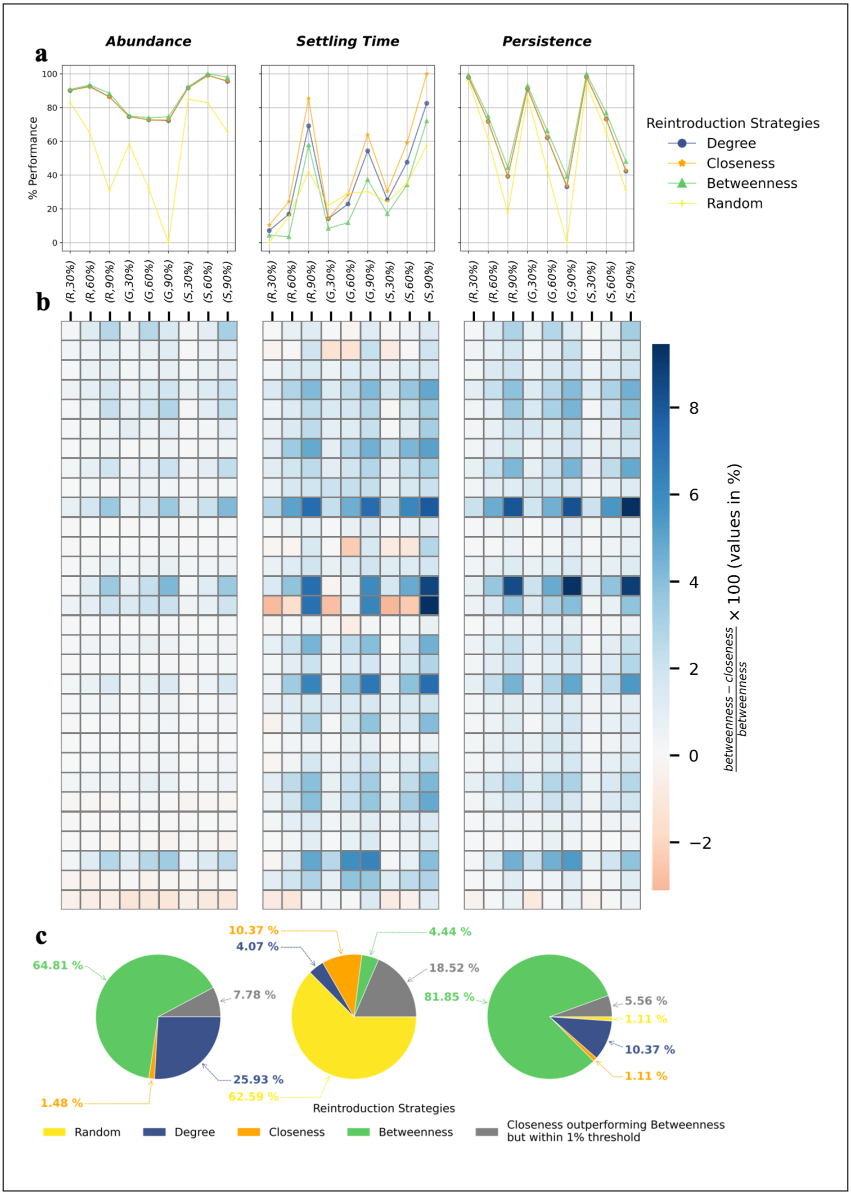

Fig. S15 Comparative Evaluation of Restoration Strategies in real-world ecosystems with respect to closeness (a) The performance of the restoration strategies measured for the three key criteria and all nine perturbation scenarios in a sample ecosystem (see 'M_PL_041' in Table S1). **(b)** This analysis is extended to the 30 real-world networks to show the relative performance of the interventions based on closeness compared to the interventions based on betweenness. **(c)** The distribution of outperforming strategies when the improvement by closeness-based interventions is within 1% of betweenness-based interventions.

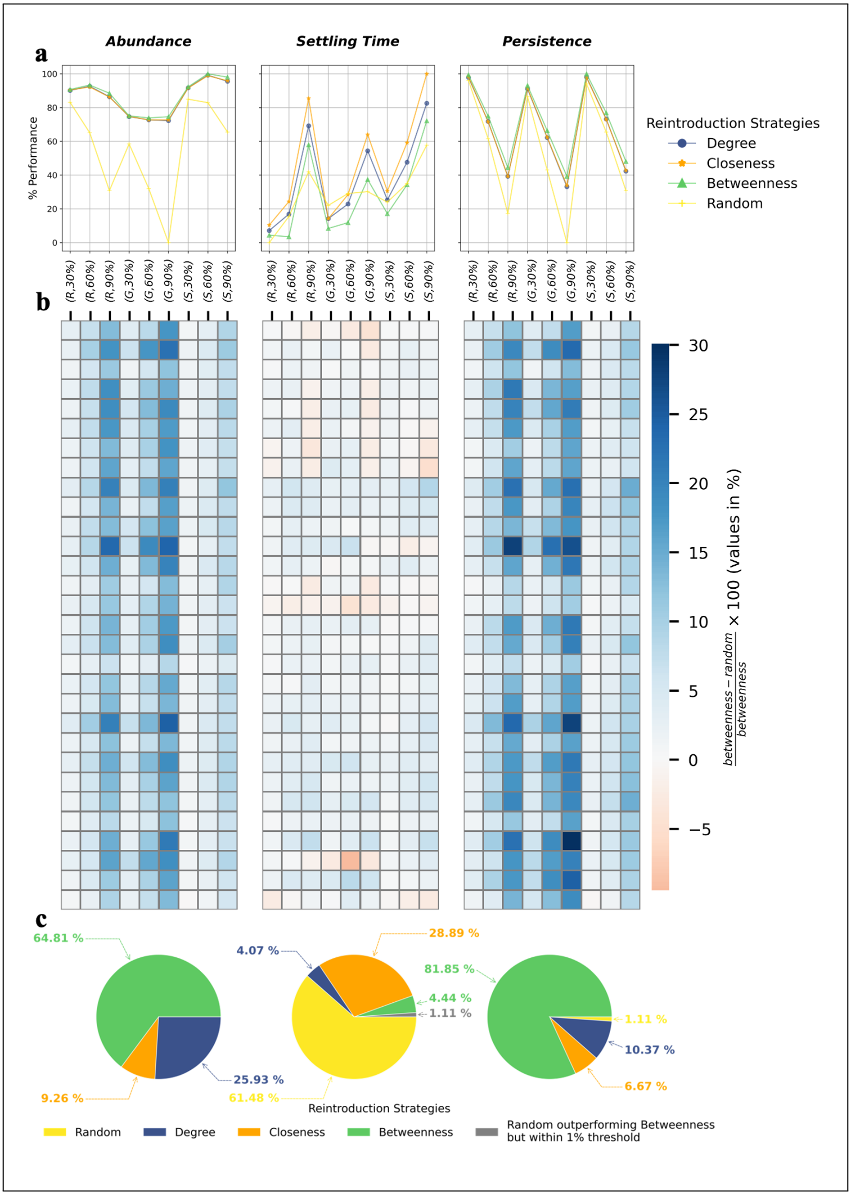

Fig. S16 Comparative Evaluation of Restoration Strategies in real-world ecosystems with respect to random (a) The performance of the restoration strategies measured for the three key criteria and all nine perturbation scenarios in a sample ecosystem (see 'M_PL_041' in Table S1). **(b)** This analysis is extended for the 30 real-world networks to show the relative performance of random interventions compared to betweenness-based interventions. **(c)** The distribution of outperforming strategies when the improvement by random interventions is within 1% of the interventions based on the distance.

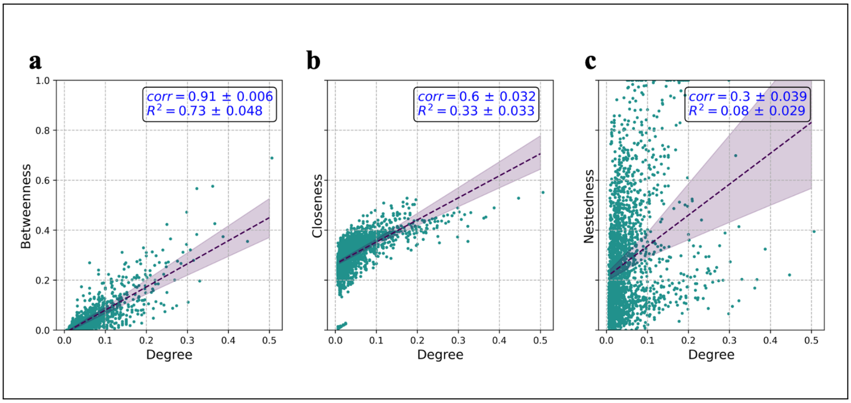

Fig. S17 The pairwise correlations of betweenness centrality **(a)**, closeness centrality **(b)**, and nestedness **(c)** with degree centrality for the 30 real-world networks. The correlations were calculated on a 95% confidence interval using 1000 bootstrap samples, each of size 500.

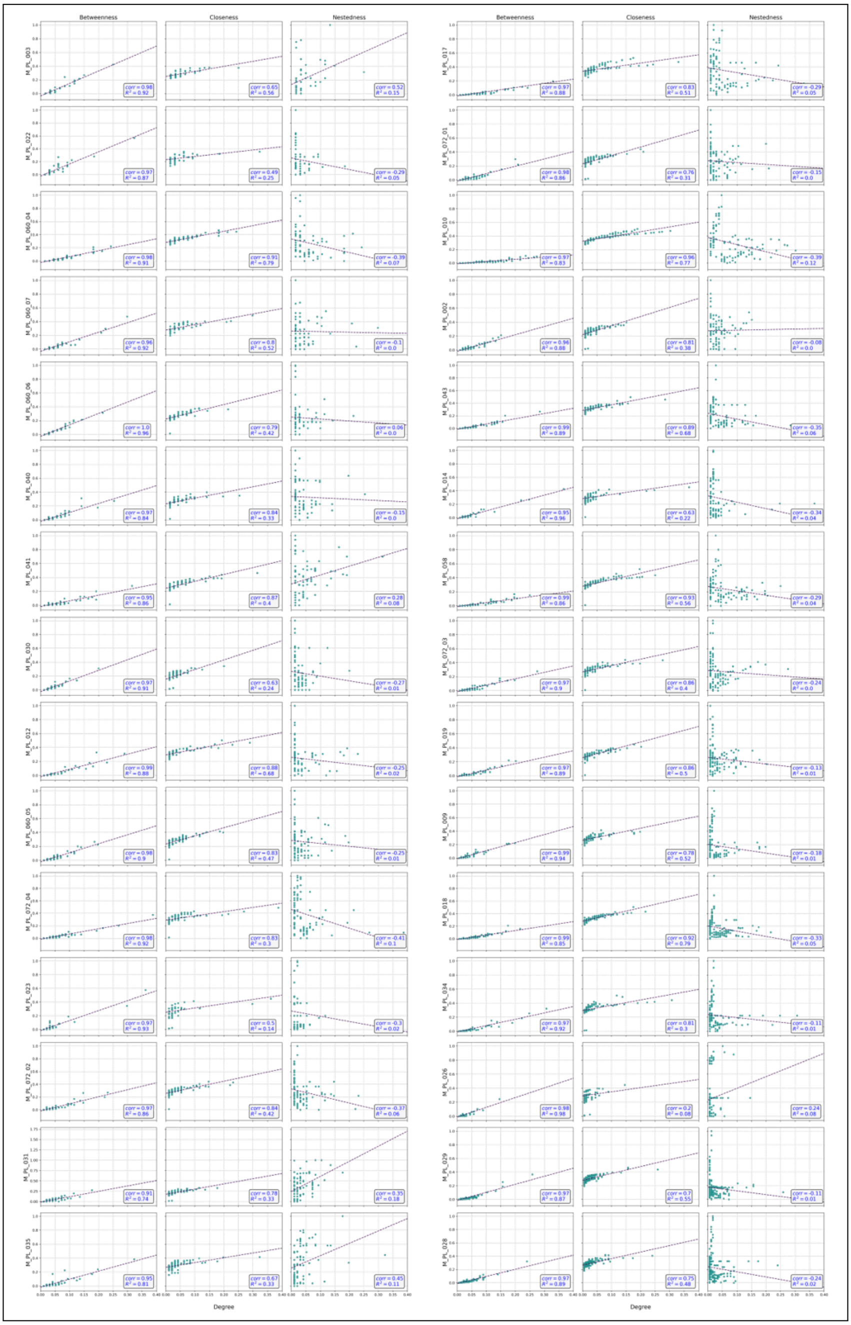

Fig. S18 The pairwise correlations of betweenness centrality, closeness centrality, and nestedness with degree centrality with corresponding Spearman rank correlation and R^2 values are shown for each of the 30 real-world networks.

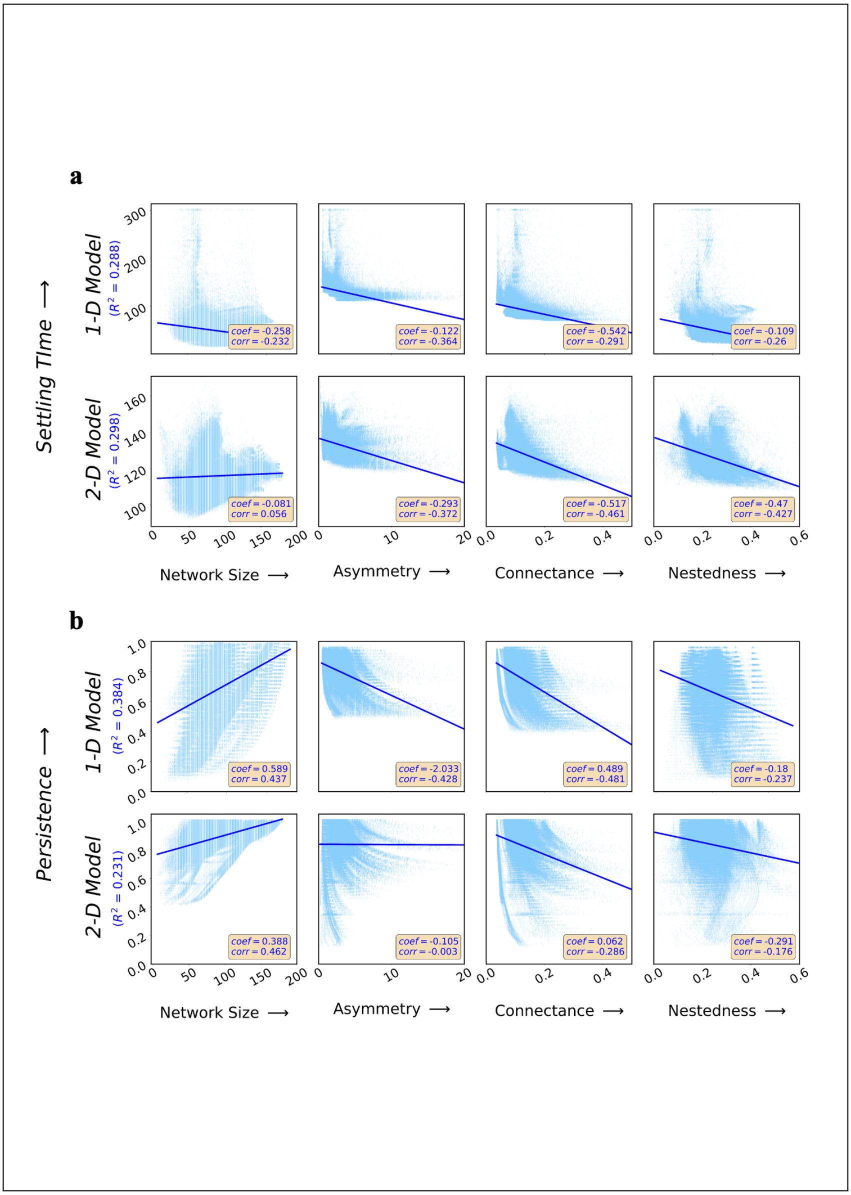

Fig. S19 The settling time **(a)** and persistence **(b)** of the surviving species is obtained after each reintroduction for every perturbed scenario considered. The panels include the Spearman rank correlation ('corr') as well as the coefficient of a multiple linear regression ('coef') and the coefficient of determination R^2 using normalized values of the network structural properties.

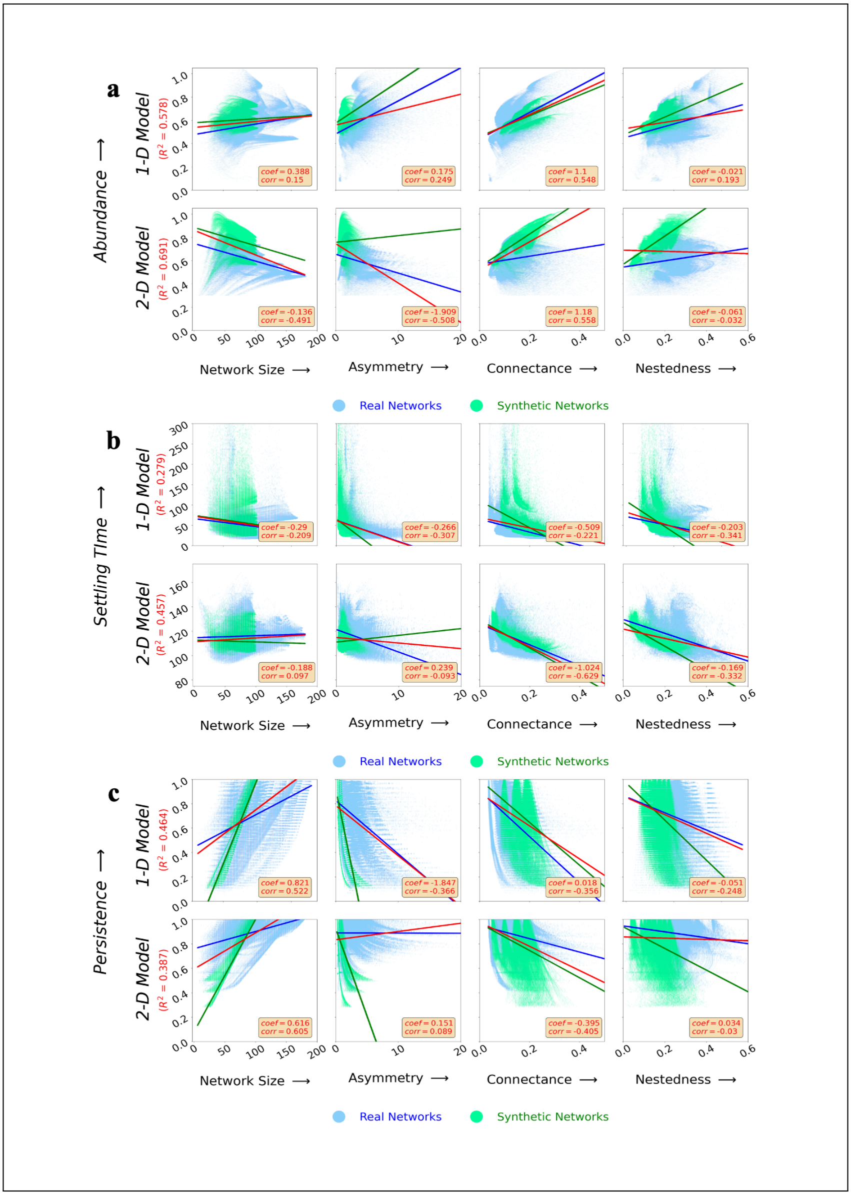

Fig. S20 The mean abundance **(a)**, settling time **(b)** and persistence **(c)** of the surviving species is obtained after each reintroduction for every perturbed scenario considered. The panels include the Spearman rank correlation ('corr') as well as the coefficient of a multiple linear regression ('coef') and the coefficient of determination R^2 using normalized values of the network structural properties. The best-fit line and the panel annotations shown in red when both real-world and synthetic networks are considered, while blue and green represent the real-world and synthetic networks respectively.

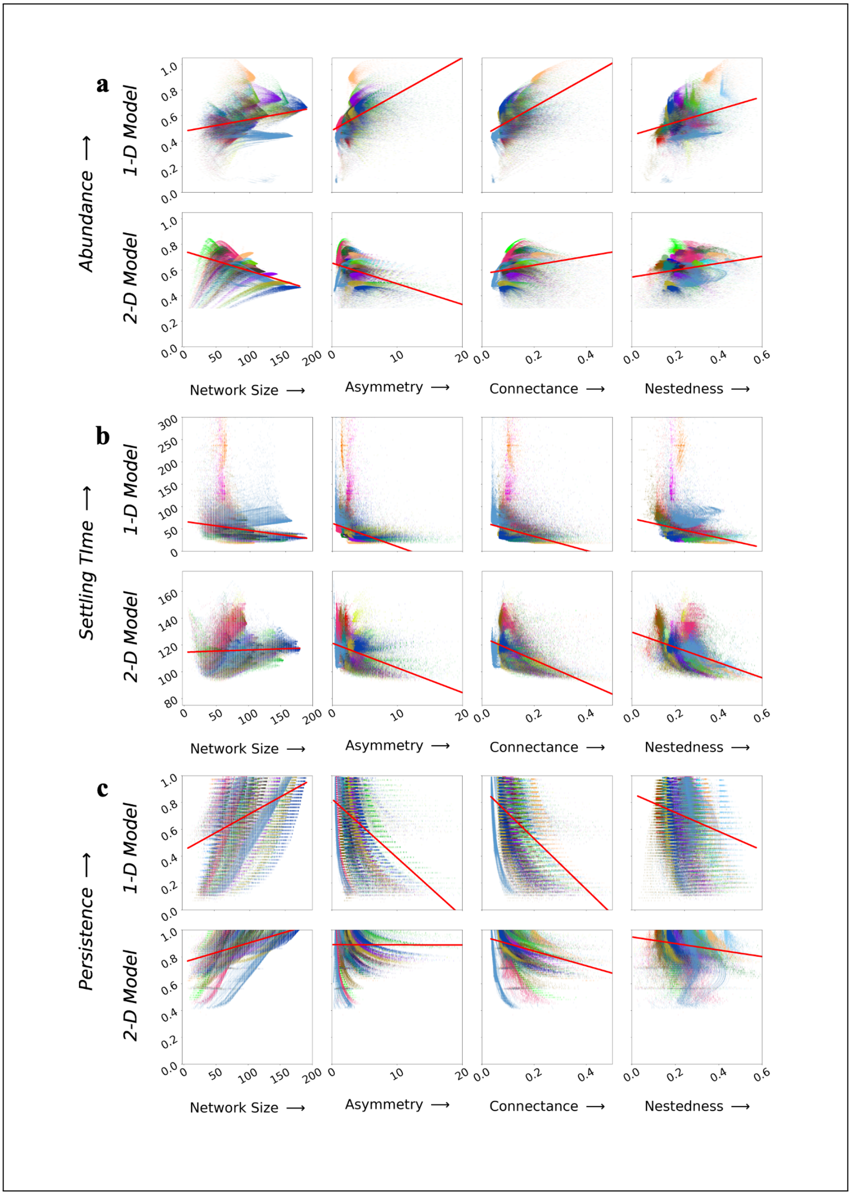

Fig. S21 The mean abundance **(a)**, settling time **(b)** and persistence **(c)** of the surviving species is obtained after each reintroduction for every perturbed scenario considered. The restoration steps observed for each of the 30 real-world networks are shown in a different color.

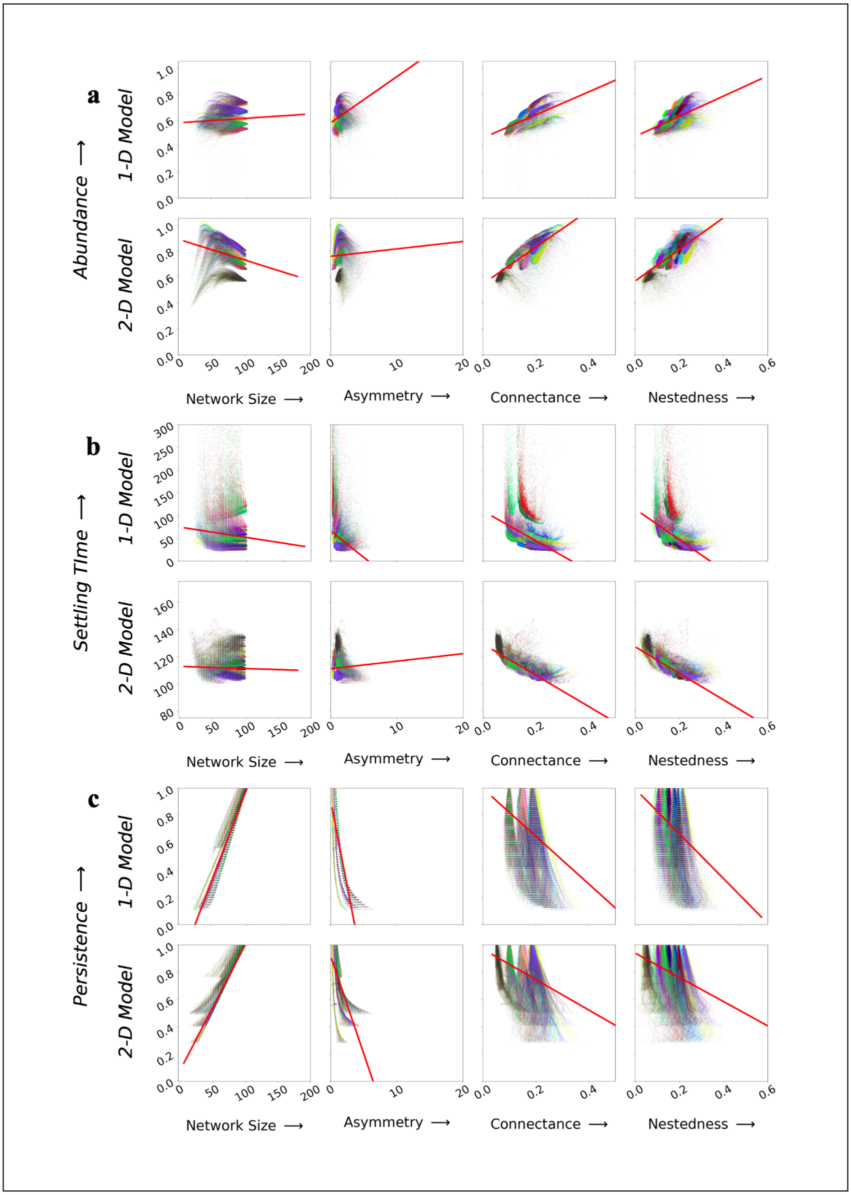

Fig. S22 The mean abundance **(a)**, settling time **(b)** and persistence **(c)** of the surviving species is obtained after each reintroduction for every perturbed scenario considered. The restoration steps observed for each of the 27 synthetic networks are shown in a different color.

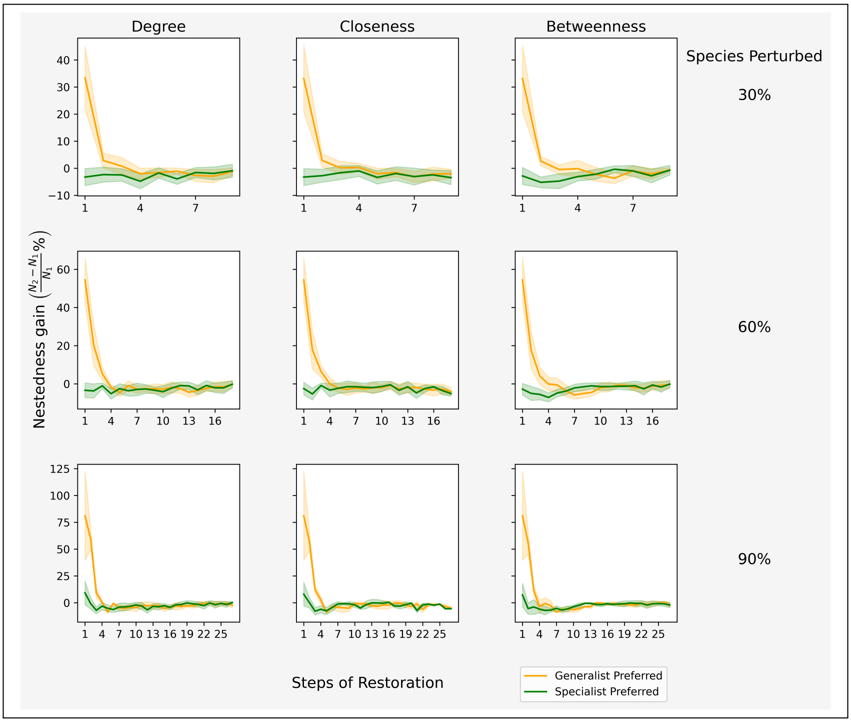

Fig. S23 The increase in nestedness resulting from sequentially reintroducing species in an example ecosystem following perturbation is shown here. The species were removed using generalist-preferred criteria, while reintroduction was performed through network-centric approaches.
