# Supplementary material for: Network-based restoration strategies maximize ecosystem recovery
Source: Commun Biol. 2023 Dec 12;6:1256. doi: 10.1038/s42003-023-05622-3 (PMC10716433; doi:10.1038/s42003-023-05622-3)
Supplement: Supplementary file 3 — Description of Supplementary Materials [file 42003_2023_5622_MOESM3_ESM.docx]

**Description of Additional Supplementary Files**

**File name:** Supplementary Data 1

**Description:** Source Data behind Fig. 1c, and 1e

**File name:** Supplementary Data 2

**Description:** Source Data behind Fig. 2a-b

**File name:** Supplementary Data 3

**Description:** Source Data behind Fig. 3b
