# Supplementary material for: Network-based restoration strategies maximize ecosystem recovery
Source: Commun Biol. 2023 Dec 12;6:1256. doi: 10.1038/s42003-023-05622-3 (PMC10716433; doi:10.1038/s42003-023-05622-3)
Supplement: Supplementary file 7 — Reporting Summary [file 42003_2023_5622_MOESM7_ESM.pdf]

Corresponding author(s): Udit Bhatia
Last updated by author(s): 2023-21-11

Reporting Summary

Nature Portfolio wishes to improve the reproducibility of the work that we publish. This form provides structure for consistency and transparency in reporting. For further information on Nature Portfolio policies, see our Editorial Policies and the Editorial Policy Checklist.

Statistics

For all statistical analyses, confirm that the following items are present in the figure legend, table legend, main text, or Methods section.

n/a	Confirmed	
☒	☐	The exact sample size (*n*) for each experimental group/condition, given as a discrete number and unit of measurement
☒	☐	A statement on whether measurements were taken from distinct samples or whether the same sample was measured repeatedly
☒	☐	The statistical test(s) used AND whether they are one- or two-sided<br>*Only common tests should be described solely by name; describe more complex techniques in the Methods section.*
☐	☒	A description of all covariates tested
☒	☐	A description of any assumptions or corrections, such as tests of normality and adjustment for multiple comparisons
☒	☐	A full description of the statistical parameters including central tendency (e.g. means) or other basic estimates (e.g. regression coefficient) AND variation (e.g. standard deviation) or associated estimates of uncertainty (e.g. confidence intervals)
☒	☐	For null hypothesis testing, the test statistic (e.g. *F*, *t*, *r*) with confidence intervals, effect sizes, degrees of freedom and *P* value noted<br>*Give P values as exact values whenever suitable.*
☒	☐	For Bayesian analysis, information on the choice of priors and Markov chain Monte Carlo settings
☒	☐	For hierarchical and complex designs, identification of the appropriate level for tests and full reporting of outcomes
☐	☒	Estimates of effect sizes (e.g. Cohen's *d*, Pearson's *r*), indicating how they were calculated

Our web collection on statistics for biologists contains articles on many of the points above.

Software and code

Policy information about availability of computer code

Data collection	Data used in this study is manually downloaded from the website www.web-of-life.es
Data analysis	All analysis is done using Python 3.11.

For manuscripts utilizing custom algorithms or software that are central to the research but not yet described in published literature, software must be made available to editors and reviewers. We strongly encourage code deposition in a community repository (e.g. GitHub). See the Nature Portfolio guidelines for submitting code & software for further information.

Data

Policy information about availability of data

All manuscripts must include a data availability statement. This statement should provide the following information, where applicable:

- Accession codes, unique identifiers, or web links for publicly available datasets
- A description of any restrictions on data availability
- For clinical datasets or third party data, please ensure that the statement adheres to our policy

Data for 30 mutualistic plant-pollinator networks were obtained from the Web of Life database (\url{www.web-of-life.es}). Data used to generate the figures are available in https://doi.org/10.5281/zenodo.10118200

Research involving human participants, their data, or biological material

Policy information about studies with human participants or human data. See also policy information about sex, gender (identity/presentation), and sexual orientation and race, ethnicity and racism.

Reporting on sex and gender	This research does not involve human participants, their data or biological material
Reporting on race, ethnicity, or other socially relevant groupings	This research does not involve human participants, their data or biological material
Population characteristics	This research does not involve human participants, their data or biological material
Recruitment	This research does not involve human participants, their data or biological material
Ethics oversight	This research does not involve human participants, their data or biological material

Note that full information on the approval of the study protocol must also be provided in the manuscript.

Field-specific reporting

Please select the one below that is the best fit for your research. If you are not sure, read the appropriate sections before making your selection.

☐ Life sciences ☐ Behavioural & social sciences ☒ Ecological, evolutionary & environmental sciences

For a reference copy of the document with all sections, see nature.com/documents/nr-reporting-summary-flat.pdf

Ecological, evolutionary & environmental sciences study design

All studies must disclose on these points even when the disclosure is negative.

Study description	Data for 30 mutualistic plant-pollinator networks were obtained from the Web of Life database (\url{www.web-of-life.es}). Data used to generate the figures are available in https://doi.org/10.5281/zenodo.10118200
Research sample	Data for 30 mutualistic plant-pollinator networks were obtained from the Web of Life database. www.web-of-life.es is a web service that provides a dataset of ecological interactions. The data was gathered from scientific studies, in most of the cases, published in scientific journals.
Sampling strategy	All datapoints generated while running the dynamical models are used for statistical analysis. To cover the spectrum of ecological properties that these real-world networks do not capture, we also simulated 27 synthetic networks to cover the broad spectrum of ecological properties.
Data collection	Data for 30 mutualistic plant-pollinator networks were obtained from the Web of Life database. www.web-of-life.es is a web service that provides a dataset of ecological interactions. The data was gathered from scientific studies, in most of the cases, published in scientific journals.
Timing and spatial scale	The data obtained cover the ecosystem level plant-pollinator interaction data.
Data exclusions	N/A
Reproducibility	No field experiments were involved. However, to ensure the reproducibility of the results, the dynamical equations and the values of the parameters and time steps are provided in the Methods and Supplementary Table 4, and the associated codes are provided via Zenodo repository. Source data underlying figures 1, 2 and 3 are provided in Supplementary Data 1, Supplementary Data 2, and Supplementary Data 3, respectively.
Randomization	We subject each network to perturbations using the 9 combinations of perturbation scenarios, with 10 ensembles for each combination.
Blinding	While simulating dynamics of pristine ecosystems, all nodes and interactions are needed to be considered. Hence, no blinding strategy is used to remove any nodes and interactions.

Did the study involve field work? ☐ Yes ☒ No

Reporting for specific materials, systems and methods

We require information from authors about some types of materials, experimental systems and methods used in many studies. Here, indicate whether each material, system or method listed is relevant to your study. If you are not sure if a list item applies to your research, read the appropriate section before selecting a response.

Materials & experimental systems

n/a	Involved in the study
☒	☐ Antibodies
☒	☐ Eukaryotic cell lines
☒	☐ Palaeontology and archaeology
☒	☐ Animals and other organisms
☒	☐ Clinical data
☒	☐ Dual use research of concern
☒	☐ Plants

Methods

n/a	Involved in the study
☒	☐ ChIP-seq
☒	☐ Flow cytometry
☒	☐ MRI-based neuroimaging

Plants

Seed stocks	n/a
Novel plant genotypes	n/a
Authentication	n/a
